# Congenital Vomer Agenesis: A Case Report From Saudi Arabia

**DOI:** 10.7759/cureus.68536

**Published:** 2024-09-03

**Authors:** Sham T Alshammeri, Abdulrhman M Almjhad, Lama B Alateeq, Ghada Alsowailmi, Mohamed Asiri, Sameer Bakhaly

**Affiliations:** 1 Medicine, University of Hail College of Medicine, Hail, SAU; 2 Medicine, Arabian Gulf University, Riyadh, SAU; 3 Otolaryngology - Head and Neck Surgery, King Abdulaziz Medical City Riyadh, Riyadh, SAU

**Keywords:** nasal septum, congenital, septal malformation, turbinate hypertrophy, vomer bone

## Abstract

The nasal septum consists of the quadrangular septal cartilage, the maxillary and palatine bone crests, the perpendicular plate of the ethmoid bone, and the vomer. Congenital agenesis of the vomer is a rare condition. This case involves a 29-year-old male who presented to our clinic with alternating bilateral nasal obstruction and no history of allergic symptoms. Examination revealed clear bilateral osteomeatal complexes without polyposis or discharge. A CT scan of the sinuses showed a right-sided septal spur and confirmed the absence of the vomer bone. This case was reported at King Abdulaziz Medical City, Riyadh, Saudi Arabia.

## Introduction

Congenital vomer agenesis (VA) is an exceptionally rare condition that can result from trauma, infections, inhalant irritants, or neoplasms [1]. VA is classified into two types based on the extent of aplasia: Type 1 involves the complete absence of the vomer from the middle turbinate to the choanae, while Type 2, or partial aplasia, affects only the caudal part of the vomer bone [2]. We report a case in which congenital VA was identified during an endoscopic examination.

## Case presentation

A 29-year-old male with no significant medical or surgical history presented to the ENT clinic with nasal obstruction. The patient reported alternating bilateral nasal obstruction with intermittent clear rhinorrhea. He denied any history of postnasal drip, hyposmia, epistaxis, facial pain, headache, allergic symptoms, or visual symptoms. He had used xylometazoline sprays intermittently over the past two years, with only partial improvement. Nasal obstruction was his only symptom, and VA was an incidental finding with no clinical relevance to his complaint.

**Figure 1:**
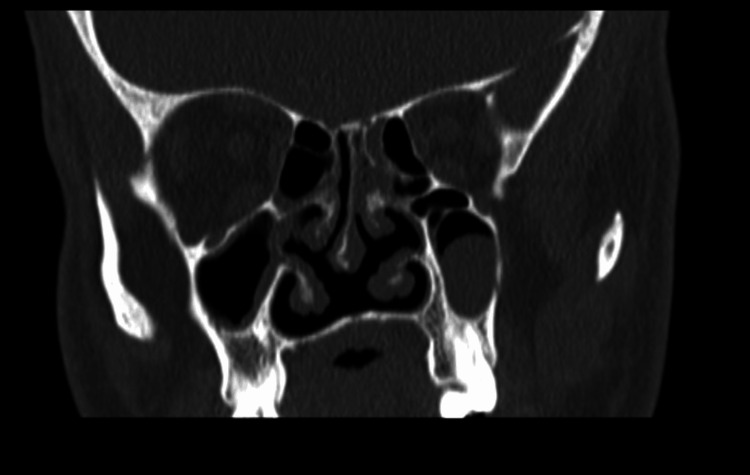
CT scan of the paranasal sinus, coronal view, showing a right-sided septal spur

**Figure 2:**
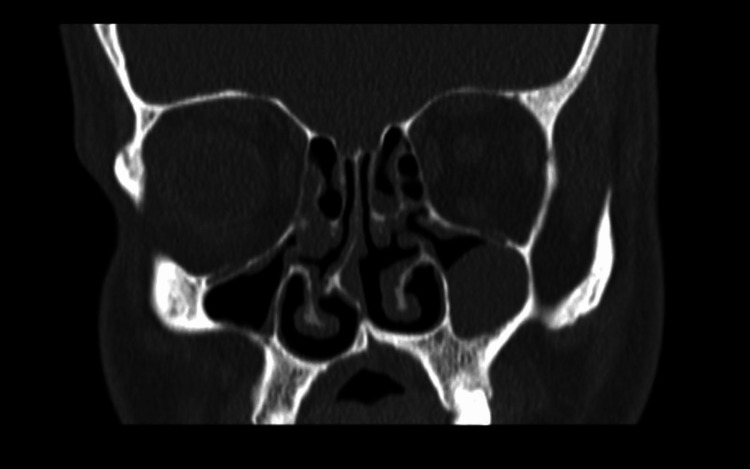
CT scan of the paranasal sinus, coronal view, showing a posterior-inferior defect in the bony septum, suggesting VA VA, vomer agenesis

**Figure 3:**
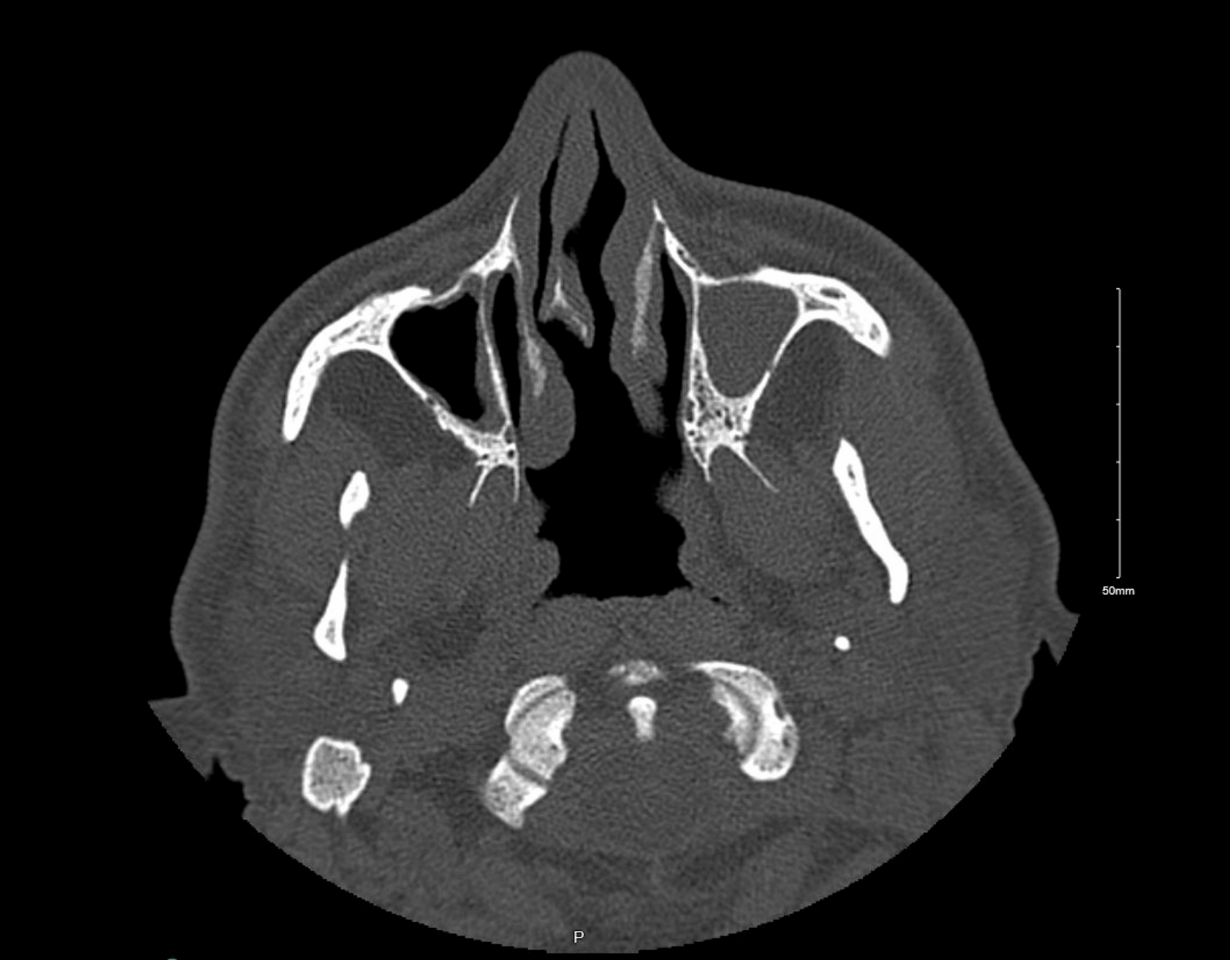
CT scan of the paranasal sinus, axial view, showing a posterior-inferior defect in the bony septum, suggesting VA VA, vomer agenesis

A zero-degree rigid endoscope examination revealed anterior right-sided nasal septal deviation, a deficient posterior nasal septum, and bilateral inferior turbinate hypertrophy. The examination showed clear bilateral osteomeatal complexes with no evidence of polyposis or discharge. A CT sinus scan was ordered, which demonstrated a right-sided septal spur and evident VA (Figure 1, Figure 2). Figure 3 showed a posterior-inferior defect of the bony septum, suggesting VA. The patient underwent conservative endoscopic septoplasty to prevent symptom progression. Postoperatively, he did well and experienced an improved functional status.

## Discussion

The vomer bone develops through three prenatal phases. The first phase, starting in the eighth week of gestation, involves intramembranous ossification in the cartilaginous nasal capsule, forming two ossification centers in the nasal septum. In the second phase, around 17 weeks, these ossified areas merge to create a U-shaped structure. By the third phase, after 17 weeks, the bones fuse in the lower part of the U, forming a Y-shaped structure [3]. Congenital VA may result from abnormalities in this developmental process. The otolaryngology department at King Abdulaziz Medical City has documented several congenital cases. Choanal atresia, a related congenital condition, involves complete or partial blockage of the nasal cavity’s posterior orifices.

Understanding the development of the vomer from prenatal stages to maturation is crucial to comprehending the etiology of congenital VA [4]. Individuals with nasal septum abnormalities often also have cleft palates and craniofacial deformities [2,5]. In cases of isolated VA, a rare anomaly, incomplete vomer development is a characteristic associated with submucosal classic cleft palate [6]. To date, approximately 22 cases of VA have been reported.

Symptoms of VA vary among affected individuals. Some are asymptomatic, with the condition discovered incidentally during routine evaluations for cough or sore throat, while others present with nasal obstruction, rhinorrhea, postnasal drip, and difficulty breathing through the nose. VA is often an incidental finding and typically does not cause symptoms unless accompanied by other conditions, such as a cleft palate.

Our patient was primarily asymptomatic, with nasal obstruction being the main complaint. Al-Shahrani and Alshehri [7] described a 35-year-old man with bilateral nasal obstruction, similar to our patient’s alternating bilateral nasal obstruction and intermittent clear rhinorrhea. Otologic symptoms have also been reported, including cases of otitis media and cholesteatoma [2,8]. Yilmaz and Altuntaş [9] documented a 19-year-old male with bilateral otitis media with effusion, hypertrophy of the posterior turbinates, septal deviation, and adenoid enlargement. Hypertrophy of the inferior turbinates is a common finding [1,2,9], as observed in our patient. Lee reported VA in conjunction with other sinonasal conditions, such as rhinosinusitis and nasal septal deviation, consistent with our case [4]. Additionally, some individuals with VA have non-otolaryngological conditions. For example, Mohri and Amatsu reported a 39-year-old male with both VA and a pituitary adenoma [8].

## Conclusions

To the best of our knowledge, only a few cases of isolated VA have been reported in the literature. Due to the absence of specific accompanying clinical presentations, VA can be easily missed or mistaken for septal perforation. As congenital VA is a rare disorder, a thorough history and physical examination are essential to rule out alternative causes of septal defects.
